# Effects of rifampin, cyclosporine A, and probenecid on the pharmacokinetic profile of canagliflozin, a sodium glucose co-transporter 2 inhibitor, in healthy participants 

**DOI:** 10.5414/CP202158

**Published:** 2014-11-19

**Authors:** Damayanthi Devineni, Nicole Vaccaro, Joe Murphy, Christopher Curtin, Rao N.V.S. Mamidi, Sveta Weiner, Shean-Sheng Wang, Jay Ariyawansa, Hans Stieltjes, Ewa Wajs, Nicholas A. Di Prospero, Paul Rothenberg

**Affiliations:** 1 Janssen Research and Development, LLC, Raritan, New Jersey,; 2 Janssen Research and Development, LLC, San Diego, California, USA, and; 3 Janssen Research and Development, A Division of Janssen Pharmaceutica NV, Turnhoutseweg, Beerse, Belgium

**Keywords:** drug-drug interactions, pharmacokinetics, UGT, MRP, P-glycoprotein

## Abstract

Objective: Canagliflozin, a sodium-glucose co-transporter 2 inhibitor, approved for the treatment of type-2 diabetes mellitus (T2DM), is metabolized by uridine diphosphate-glucuronosyltransferases (UGT) 1A9 and UGT2B4, and is a substrate of P-glycoprotein (P-gp). Canagliflozin exposures may be affected by coadministration of drugs that induce (e.g., rifampin for UGT) or inhibit (e.g. probenecid for UGT; cyclosporine A for P-gp) these pathways. The primary objective of these three independent studies (single-center, open-label, fixed-sequence) was to evaluate the effects of rifampin (study 1), probenecid (study 2), and cyclosporine A (study 3) on the pharmacokinetics of canagliflozin in healthy participants. Methods: Participants received; in study 1: canagliflozin 300 mg (days 1 and 10), rifampin 600 mg (days 4 – 12); study 2: canagliflozin 300 mg (days 1 – 17), probenecid 500 mg twice daily (days 15 – 17); and study 3: canagliflozin 300 mg (days 1 – 8), cyclosporine A 400 mg (day 8). Pharmacokinetics were assessed at pre-specified intervals on days 1 and 10 (study 1); on days 14 and 17 (study 2), and on days 2 – 8 (study 3). Results: Rifampin decreased the maximum plasma canagliflozin concentration (C_max_) by 28% and its area under the curve (AUC) by 51%. Probenecid increased the C_max_ by 13% and the AUC by 21%. Cyclosporine A increased the AUC by 23% but did not affect the C_max_. Conclusion: Coadministration of canagliflozin with rifampin, probenecid, and cyclosporine A was well-tolerated. No clinically meaningful interactions were observed for probenecid or cyclosporine A, while rifampin coadministration modestly reduced canagliflozin plasma concentrations and could necessitate an appropriate monitoring of glycemic control.

## Introduction

Pharmacotherapeutic management of type-2 diabetes mellitus (T2DM) is based on the severity of disease, and the efficacy and tolerability of the therapeutic agents. Currently, oral hypoglycemic agents or insulin (if required) are commonly used for T2DM treatment [1, 2, 3]. However, due to poor glycemic control [4] and adverse effects (such as hypoglycemia and weight gain) of existing drugs, there is a need for new pharmacologic agents that can be either used as a monotherapy or in combination with existing medications [2, 5]. 

Canagliflozin (Invokana™), a novel selective sodium glucose co-transporter 2 (SGLT2) inhibitor, is approved in many countries around the world at doses of 100 and 300 mg once daily (q.d.) as an adjunct to diet and exercise to improve glycemic control in adults with T2DM [6, 7, 8]. Canagliflozin inhibits renal SGLT2 activity, which decreases renal glucose reabsorption, thereby increasing urinary glucose excretion (UGE), and decreasing plasma glucose levels [2, 5, 9]. Canagliflozin treatment is also associated with meaningful decrease in body weight consistent with urinary calorie loss (as glucose) [9, 10, 11]. 

Canagliflozin is primarily metabolized to two pharmacologically inactive O-glucuronides (M7 and M5), by uridine diphosphate-glucuronosyltransferase (UGT) 1A9 and UGT2B4 enzymes, respectively [12, 13, 14], while cytochrome P450 (CYP450) 3A4 plays a minimal role in its metabolism [14]. Additionally, in vitro experiments have indicated that canagliflozin is a substrate of P-glycoprotein (P-gp) and multidrug resistance protein 2 (MRP2) [6]. 

Both UGT1A9 and UGT2B4 enzymes were reported to be inducible in humans [15, 16]. Because canagliflozin is metabolized by UGT1A9 and UGT2B4, induction of these enzymes may lead to lower plasma canagliflozin concentrations and reduced pharmacodynamic efficacy. Hence, the effects of rifampin (antitubercular drug), a prototypical UGT inducer, on canagliflozin pharmacokinetics (PK) were evaluated. Rifampin was used as it potently induces several UGT enzymes (UGT1A1, UGT1A4, UGT1A9, UGT2B4, and UGT2B7), CYP450 isozymes (CYP3A4, CYP2C8, and CYP2C9) as well as some drug transporters (including P-gp and MRP2) [17, 18, 19, 20, 21]. 

Probenecid, a frequently used anti-gout medication [22], is a general in vivo inhibitor of UGT enzymes (UGT1A1, UGT1A6, UGT1A7, UGT1A9, UGT1A10, and UGT2B7) [23]. Additionally, it inhibits several drug transporters, including MRP2, organic anion transporting polypeptide (OATP), and organic anion transporter families (OAT1 and OAT3) [24, 25, 26]. Because UGT inhibition may lead to increased canagliflozin plasma concentrations, it was important to determine whether coadministration of an UGT inhibitor may affect the systemic exposure of canagliflozin. Moreover, because there are no known isozyme-selective UGT inhibitors [27], probenecid was used as a probe. 

The immunosuppressant drug cyclosporine A is a potent inhibitor of the multidrug efflux transporter P-gp. Cyclosporine A also inhibits other drug-metabolizing enzymes, such as CYP3A4 and drug transporters (e.g., OATP2 and MRP2) [28]. In vitro experiments in multidrug resistant protein 1 expressed in Madin Darby canine kidney cells indicated that canagliflozin is a P-gp substrate with a basal-to-apical/apical-to-basal efflux ratio of 2.0. The efflux was inhibited (80%) in the presence of cyclosporine A at a concentration of 10 µM (unpublished data). Hence, it was important to investigate the potential effects of a potent P-gp inhibitor on canagliflozin disposition. 

This report summarizes the results of three separate studies that investigated the effect of rifampin, probenecid, and cyclosporine A on the PK of canagliflozin in healthy participants. 

## Methods

### Study population

Healthy men and women, between 18 and 55 years of age, with body mass index (BMI) between 18 and 30 kg/m^2^ and body weight of ≥ 50 kg, who were healthy based on medical history, physical examination, and clinical laboratory evaluations, were enrolled in all the three studies. Included were women who were postmenopausal, surgically sterile, or women of child-bearing potential who had a negative serum β-human chorionic gonadotropin pregnancy test and men, who agreed to use an adequate contraception method. Individuals with a history of drug or alcohol abuse were excluded. Participants refrained from taking any drugs or substances known to inhibit or induce CYP450 enzymes and/or P-gp within 28 days prior to the first dose and throughout the study period. In the probenecid study, participants with a history of, or current acute or chronic renal insufficiency (expected glomerular filtration rate (eGFR): < 90 mL/min/1.73 m^2^) kidney or bladder stones (nephrolithiasis), hyperuricosuria (> 800 mg/day), gout or hyperuricemia (> 6.8 mg/dL) were excluded. Participants were not allowed to take concomitant medications throughout the study period. 

The protocol for each of the studies was approved by an Independent Ethics Committee or the Institutional Review Board at each study site, and the studies were conducted in accordance with the ethical principles originating in the Declaration of Helsinki and in accordance with the International Conference on Harmonization (ICH) Good Clinical Practice guidelines, applicable regulatory requirements, and in compliance with the protocol. All participants provided written informed consent to participate in the studies. 

### Study designs and treatment

**Figure 1.**
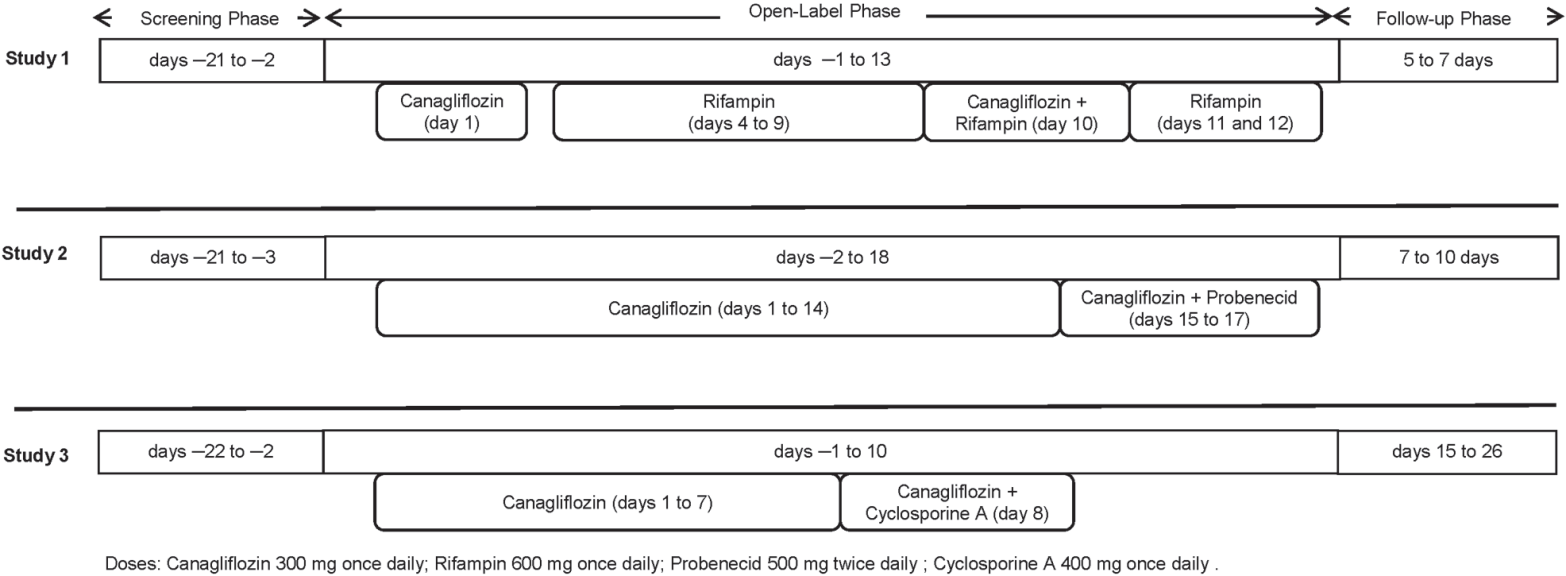
Study design.

The study designs for all the three studies are presented in Figure 1. For each study, the participants were enrolled at different individual study centers in the US. Study 1 (rifampin study, NCT01395927) was a single-center, open-label, fixed-sequence study to evaluate the effects of steady-state rifampin on the single-dose PK of canagliflozin. The study consisted of a screening period of 20 days (from day –21 to ‒2), a 14-day open-label period (from day –1 to 13), followed by an end-of-study period (5 – 7 days) consisting of follow-up assessments. Participants received canagliflozin 300 mg (tablet) q.d. on day 1 and rifampin 600 mg q.d. (as two 300 mg capsules) on days 4 – 9. On day 10, canagliflozin (300 mg) plus rifampin (600 mg) was administered, followed by rifampin alone (600 mg) administration on days 11 – 12. The study drugs were administered under a fasted state. 

Study 2 (probenecid study, NCT01428284) was a single-center, open-label, fixed-sequence study to evaluate the effects of multiple-dose probenecid on the steady-state PK of canagliflozin. The study consisted of a 19-day screening period (from day –21 to –3), a 20 day open-label period (from day –2 to 18), followed by an end-of-study period (7 – 10 days). Canagliflozin 300 mg (tablet) q.d. was administered on days 1 through 14, followed by canagliflozin 300 mg q.d. plus probenecid 500 mg (tablet) twice daily (b.i.d.) on days 15 through 17. Participants received study drugs under fasted state on days 14 and 17. On all other study days, participants received a standardized meal 1 hour after the study drug administration. Diet was standardized in order to stabilize uric acid levels, and participants were advised not to consume highly purine-enriched foods. 

Study 3 (cyclosporine A study, NCT01718652) was a single-center, open-label, fixed-sequence study to evaluate the effects of a single dose of cyclosporine A on the steady-state PK of canagliflozin. The study consisted of a 3-week screening period (from day –22 to –2), an 11 day open-label period (from day –1 to 10), followed by an end-of-study period consisting of 2 follow-up assessments occurring from 7 to 10 days and again from 14 to 18 days after the last dose of the study drug administration. Canagliflozin 300 mg (tablet) q.d. was administered on days 1 through 8 under fasted state. On day 8, a single oral dose of cyclosporine A (400 mg capsule) was administered 30 minutes before the final dose of canagliflozin. 

### Pharmacokinetic evaluations

**Sample collection**

Study 1: Venous blood (4 mL each) samples for the determination of canagliflozin, M7, and M5 plasma concentrations were collected pre-dose, and up to 72 hours post-dose (at 0.5, 1, 1.5, 2, 3, 4, 6, 8, 10, 12, 16, 24, 48, and 72 hours) on days 1 and 10. Urine samples for the determination of canagliflozin, M7, and M5 in urine were collected over the intervals of 0 – 4 hours, 4 – 12 hours, 12 – 24 hours, 24 – 48 hours, and 48 – 72 hours on days 1 and 10. Venous blood samples (2 mL each) for the determination of rifampin plasma concentrations were collected 2 hours after rifampin administration on days 9 and 10. 

Study 2: Venous blood (4 mL each) samples for the determination of canagliflozin, M7, and M5 plasma concentrations were collected pre-dose and up to 24 hours postdose (at 0.5, 1, 1.5, 2, 3, 4, 6, 8, 10, 12, 16, and 24 hours) on days 14 and 17. Urine samples for the determination of canagliflozin, M7, and M5 in urine were collected over the intervals of 0 – 4 hours, 4 – 12 hours, and 12 – 24 hours on days 14 and 17. Venous blood samples (2 mL each) for the determination of probenecid plasma concentrations were collected 2 hours after probenecid administration (the morning dose) on days 15 – 17. 

Study 3: Venous blood (4 mL each) samples for the determination of canagliflozin plasma concentration were collected at pre-dose on days 2, 3, 4, 5, and 6, and at pre-dose and up to 24 hours post-dose (at 0.5, 1, 1.5, 2, 3, 4, 5, 6, 10, 12, 16, and 24 hours) on days 7 and 8. A single blood sample (2 mL) for the determination of blood concentration of cyclosporine A was collected 2.5 hours after cyclosporine A administration on day 8. 

Blood samples for the PK evaluation were collected in the following collection tubes: canagliflozin, M7, and M5: dipotassium ethylenediaminetetraacetic acid (K_2_EDTA); rifampin and probenecid: sodium-heparin; and cyclosporine A: EDTA. Blood samples for canagliflozin, M7 and M5, rifampin and probenecid were centrifuged (10 minutes at 1,300 rpm), and the obtained plasma was stored at or below –20 ºC. For cyclosporine A, within 20 minutes of collection, an aliquot of 0.8 mL of blood was transferred to a pre-labeled storage container already containing 0.8 mL of water, after which the samples were thoroughly mixed by shaking, and the water-diluted blood samples were stored at or below –20 ºC. Urine samples were collected in polyethylene containers for the PK evaluation of canagliflozin, M7, and M5. Two aliquots (3 mL in study 1 and 2 mL in study 2) of urine sample were transferred into polyethylene tubes and were stored at or below –20 °C. 

**Analytical methods**

The EDTA plasma samples were analyzed to determine the concentrations of canagliflozin with a validated [29, 30] liquid chromatography coupled to tandem mass spectrometry/mass spectrometry (LC-MS/MS) method (Frontage, Shanghai, China). A ^13^C_6_ analogue of canagliflozin was used as internal standard (IS). Briefly, the sample was processed using a liquid-liquid extraction with tert-butyl methyl ether. The LC phase used a 5 cm × 4.6 mm column packed with XBridge C18 (Waters, Milford, MA, USA) with a mobile phase of ammonium acetate 0.01M (30%) and methanol (70%), and a flow rate of 1.2 mL/min. Quantification was achieved by MS/MS detection with an API4000, equipped with TurboIonSpray (TIS) interface (AB Sciex, Framingham, MA, USA), in the positive ion multiple reaction monitoring (MRM) mode. Canagliflozin and IS were monitored at mass transitions m/z 462.1 → 267.0 and m/z 468.1 → 273.0, respectively. The quantitation range was 5.0 – 10,000 ng/mL. Urine samples were quantified in the range of 25 – 10,000 ng/mL using a qualified assay that essentially did not deviate from the plasma method. 

Plasma (validated) and urine (qualified) concentrations of M7 and M5 were also determined using ^13^C_6_-canagliflozin as IS at Frontage Laboratories (Shanghai) Co., Ltd. Samples underwent protein precipitation, with acetonitrile. The LC-MS/MS quantitation was done with the same equipment as for canagliflozin. The LC phase used a 5 cm × 4.6 mm column packed with XBridge C18 with a mobile phase of ammonium acetate 0.01M (pH 4) and methanol at ratio 40/60 (v/v) at a flow rate of 1.2 mL/min. After 3.5 minutes, the composition was changed to 98% methanol for 1 minute to flush the column. Using positive ion MRM on an API4000, equipped with TIS, both metabolites were measured at mass transition m/z 638.2 → 427. The quantitation range was 5.0 – 10,000 ng/mL for both metabolites in plasma, and 100 – 100,000 ng/mL in urine. 

Rifampin concentrations were determined in plasma samples at PPD, Middleton, WI, USA, and cyclosporine A and probenecid concentrations were determined at PRA, Assen, The Netherlands. 

**Pharmacokinetic analyses**

Pharmacokinetic analyses of plasma and urine concentrations was done by non-compartmental method using validated WinNonlin^®^ software Version 5.2.1 (Pharsight Corporation, Mountain View, CA, USA). Pharmacokinetic parameters of canagliflozin, M7, and M5 determined in study 1 included maximum observed plasma concentration (C_max_), time to reach C_max_ (t_max_), terminal elimination half-life (t_1/2_), area under the plasma concentration-time curve from time 0 to infinite time (AUC_∞_), cumulative amount of drug excreted in urine, expressed as a percentage of the administered dose (% Ae, dose), renal clearance (CL_R_), and metabolite to parent ratio (M/P (corrected for the differences in molecular weights)) for C_max_ and AUC_∞._ The assessed pharmacokinetic parameters of canagliflozin, M7, and M5 in study 2, and of canagliflozin in study 3 were: C_max_ during a dosing interval at steady state (C_max.ss_), observed plasma concentration before dosing or at the end of a dosing interval (C_trough_; only for canagliflozin), t_max_ during a dosing interval at steady state (t_max.ss_), and AUC during a dosing interval at steady state (AUC_τ.ss_). Additionally, M/P ratio (corrected for the differences in molecular weights) for C_max.ss_, AUC_τ.ss_, Ae during a collection interval (Ae_t1-t2_), %Ae dose, and CL_R_ were calculated for study 2. 

### Safety assessments

Safety and tolerability were monitored throughout all three studies by evaluation of the incidence and the type of treatment-emergent adverse events (TEAEs), and change from baseline in: clinical laboratory tests (including hematology, serum chemistry, urinalysis, and urine uric acid analysis (study 2 only)), physical examination of all body systems except genitalia and breast (by a study physician), body weight, height, and BMI, vital signs (including systolic and diastolic blood pressure, heart rate, and body temperature), and 12-lead electrocardiographys (ECGs) (including cardiac rhythm, waveform morphology, and interval duration). 

### Sample size determination

Based on former studies with canagliflozin, the intra-participant coefficient of variation (% CV) was estimated to be ≤ 20% for AUC and C_max_; using an estimated 20% CV for AUC and C_max_ of canagliflozin, a sample size of 12 completed participants each for studies 1 and 2 would be sufficient for the ratio of mean PK parameters of canagliflozin with and without coadministration of the other drug to fall within 86% and 116%, and 16 completed participants for study 3 to fall within 88.3% and 113.3% of the true value with 90% confidence. In studies 1 and 2, a total of 14 participants were enrolled to ensure that at least 12 participants completed the study; and in study 3, 18 participants were enrolled to ensure that at least 16 participants completed the study. 

### Statistical analyses

Statistical analysis for the PK interaction of plasma canagliflozin was performed on the log-transformed values (AUC_∞_ and C_max_ for study 1; AUC_τ.ss_ and C_max.ss_ for studies 2 and 3). A mixed-effects model was fitted to the log-transformed data of AUC or C_max_ as dependent variables, treatment (with or without coadministration of the other drug) as a fixed effect, and participant as a random effect. The estimated least-square means and intra-participant variability from the mixed-effect model was used to construct 90% confidence interval (CI) for the difference in means of AUC or C_max_ on the log-scale between the two treatments. The limits of the CIs were retransformed using antilogarithms to obtain the 90% CI for the ratio of the geometric mean AUC or C_max_. 

The TEAEs were summarized by treatment within each body system and for each preferred term. Results of the clinical laboratory parameters and vital signs were summarized using descriptive statistics. 

## Results

### Participant disposition and demographics

The participant disposition and demographics and baseline characteristics for all the three studies are presented in Table 1. In study 1, 14 participants (all men) were enrolled, and all the participants completed the study. Most participants were White (n = 11; 79%), with a mean age of 30.3 years. In study 2, 14 participants were enrolled, and 11 participants completed the study. Three participants were withdrawn from the study because of lost to follow-up, protocol violation, or physician decision (1 participant each). One participant, who was lost to follow-up, completed all the required assessments and was included in both PK and statistical analyses. Most of the participants were men (n = 13; 93%), and Black or African-American (n = 9; 64%) with a mean age of 29.1 years. In study 3, 18 participants (all White) were enrolled, and all the participants completed the study. Most of the participants were men (n = 15; 83%), and the mean age of the participants was 42.1 years. 

### Study 1

**Effect of rifampin on the pharmacokinetics of canagliflozin and its inactive metabolites (M7 and M5)**

**Figure 2.**
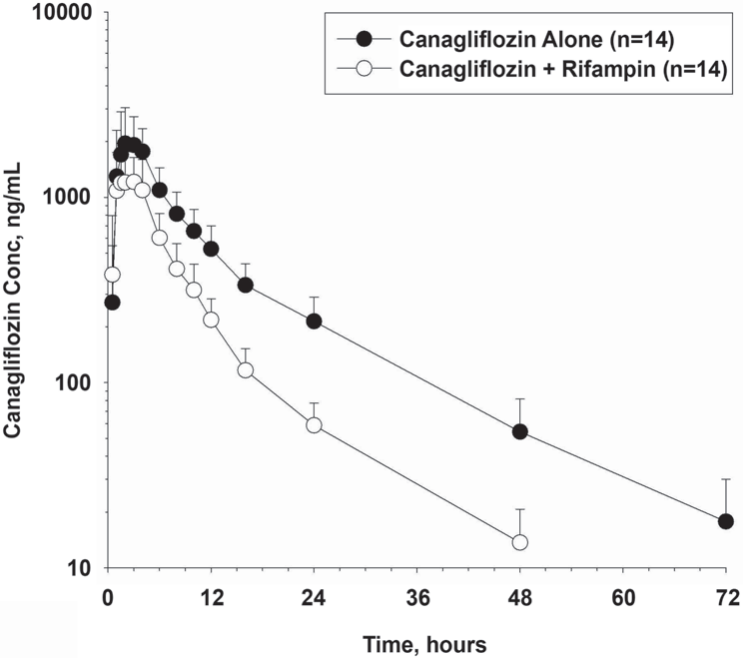
Mean (SD) canagliflozin plasma concentration-time profiles following administration of canagliflozin alone and with rifampin in healthy participants. SD = standard deviation; Conc = concentration.

Coadministration of canagliflozin with rifampin decreased the mean plasma concentrations of canagliflozin (Figure 2). The mean plasma concentrations of the metabolites M7 and M5 increased for the first few hours and then decreased over the 24-hour period, compared with canagliflozin treatment alone. Canagliflozin was rapidly absorbed; peak concentration was reached at a median time of 2 hours following administration of canagliflozin alone, which was similar to when coadministered with rifampin (median t_max_, 1.79 hour). Following rifampin coadministration, reductions in canagliflozin mean C_max_ by 28%, in AUC_∞_ by 51%, and in t_1/2_ by ~ 13% were observed compared with when canagliflozin was administered alone. The geometric mean estimated 90% CI for canagliflozin and rifampin coadministration, compared with canagliflozin alone for C_max_ was 71.75 (61.13; 84.21) and for AUC_∞_ was 48.76 (43.69; 54.43) (Table 2). Following rifampin coadministration, higher mean C_max_, M/P C_max_, M/P AUC_∞_, lower AUC_∞_, was t_1/2_, and similar CL_R_ were observed for M7 compared with when canagliflozin was administered alone. Similarly, higher mean C_max_, M/P C_max_, M/P AUC_∞_, similar AUC_∞_, t_1/2_, and CL_R_ were observed for M5 following rifampin coadministration compared with when canagliflozin was administered alone (Table 2). 

Plasma rifampin concentration at steady-state was increased (by 24%) at 2 hours following coadministration with canagliflozin under the fasted state on day 10 compared with rifampin administration alone under the fed state on day 9 (mean (standard deviation, SD): 11,323 (1,946) ng/mL vs. 9,156 (2,872) ng/mL), which is consistent with previous observations [31]. Because rifampin absorption is reduced (by 30%) under fed conditions [32, 33], this may have contributed to the observed difference in plasma rifampin concentrations. 

### Study 2

**Effect of probenecid on the pharmacokinetics of canagliflozin and its inactive metabolites (M7 and M5)**

**Figure 3.**
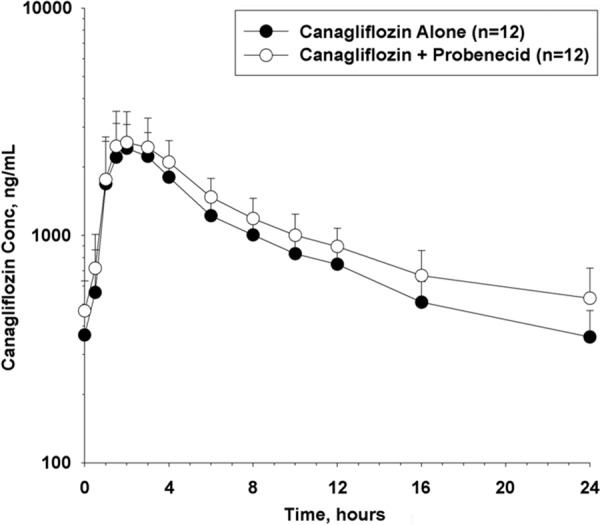
Mean (SD) canagliflozin plasma concentration-time profiles following administration of canagliflozin alone and with probenecid in healthy participants. SD = standard deviation; Conc = concentration.

Mean (SD) canagliflozin trough plasma concentrations were 381 (110) ng/mL on day 13, 366 (117) ng/mL on day 14, and 358 (110) ng/mL on day 15, indicating steady state levels. Canagliflozin was rapidly absorbed; peak concentration was reached at similar median time following administration of canagliflozin alone and when co-administered with probenecid (median t_max_: 1.92 vs. 1.67 hours) (Table 3). The mean plasma concentrations increased for canagliflozin (Figure 3), M7, and M5 after probenecid coadministration. Probenecid coadministration resulted in an ~ 13% and 21% higher canagliflozin C_max.ss_ and AUC_τ.ss_, respectively, as compared with when canagliflozin was administered alone. The geometric mean estimated 90% CI for canagliflozin and probenecid coadministration compared with canagliflozin alone for C_max.ss_ was 113.37 (100.37; 128.06) and for AUC_τ.ss_ 120.74 (116.37; 125.27). Mean canagliflozin CL_R_ was ~ 34% lower for canagliflozin coadministered with probenecid compared with canagliflozin alone (Table 3). Following probenecid coadministration, a higher mean C_max.ss_, AUC_τ.ss_, M/P C_max_, and M/P AUC_τ.ss_, and lower CL_R_ for M7 and M5 were observed, compared with when canagliflozin was administered alone (Table 3). 

Plasma probenecid concentrations (mean (SD)) at 2 hours after morning probenecid administration on days 15, 16, and 17 were 38,275 (13,849), 58,917 (15,178), and 65,275 (19,666) ng/mL, respectively. The mean (SD) plasma probenecid concentration observed on day 17 at 2 hours post-dose is consistent with published data for a 500 mg b.i.d. dosing regimen [34]. 

### Study 3

**Effect of cyclosporine A on the pharmacokinetics of canagliflozin**

**Figure 4.**
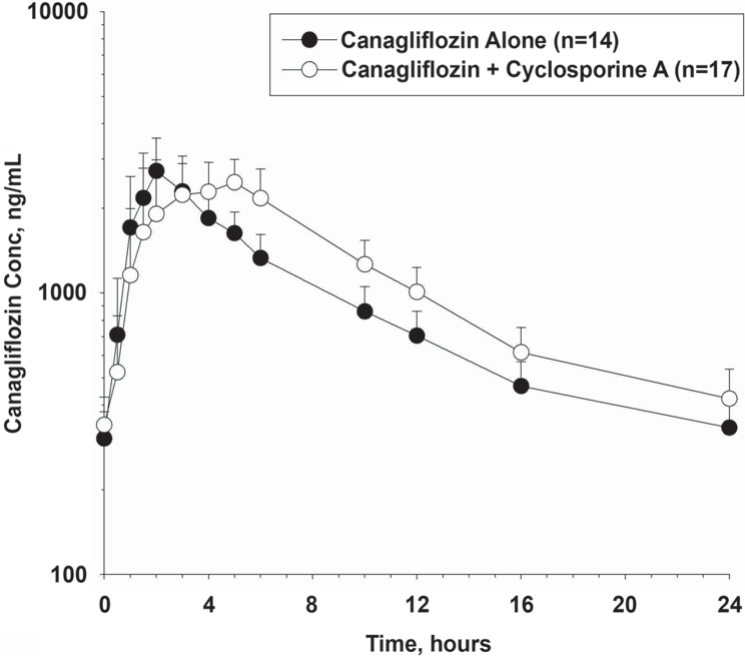
Mean (SD) canagliflozin plasma concentration-time profiles following administration of canagliflozin alone and with cyclosporine A in healthy participants. SD = standard deviation; Conc = concentration.

Mean canagliflozin trough plasma concentrations reached steady-state (mean (SD): 318 (94.2) ng/mL) by day 4, indicating that the effects of cyclosporine A on canagliflozin PK were evaluated at a point when plasma canagliflozin levels were at steady-state. Canagliflozin was rapidly absorbed; peak concentration was reached at a median time of 2 hours following administration of canagliflozin alone, and was slightly delayed when co-administered with cyclosporine A (median t_max_, 4 hours; Figure 4). Coadministration of a single dose of oral cyclosporine A with multiple doses of canagliflozin resulted in an ~ 23% increase in mean canagliflozin AUC_τ.ss_, but did not affect mean C_max.ss_. The geometric mean estimated 90% CI for canagliflozin and cyclosporine A coadministration compared with canagliflozin alone for C_max.ss_ was 100.81 (91.31; 111.30), and for AUC_τ.ss_ 122.98 (118.66; 127.46) (Table 4). 

## Safety 

Concomitant administration of canagliflozin with rifampin, probenecid, and cyclosporine A was well-tolerated, with mild TEAEs which were generally transient. 

In study 1, TEAEs were more frequent after the administration of rifampin alone (50%; 7/14) and canagliflozin alone (21.4%; 3/14) than during rifampin and canagliflozin coadministration (14.3%; 2/14). Polyuria, abdominal discomfort, and thirst were reported in 7.1% (1/14) of participants with canagliflozin alone treatment. 

In study 2, 28.6% (4/14) of participants receiving canagliflozin alone and 41.7% (5/12) of participants receiving canagliflozin and probenecid coadministration experienced ≥ 1 TEAE. Headache was the most common TEAE reported with canagliflozin alone (14.3%; 2/14), while headache, abdominal discomfort, diarrhea, muscle twitching, musculoskeletal pain, urethral pain, and pruritis were observed with canagliflozin and probenecid coadministration (8.3%; 1/12 for each TEAE). 

In study 3, more participants (94.4%; 17/18) receiving canagliflozin and cyclosporine A coadministration reported TEAEs compared with those receiving canagliflozin alone (33.3%; 6/18). Diarrhea and oropharyngeal pain were the most common TEAEs reported with canagliflozin alone (11.1%; 2/18 for each TEAE), while flushing (a known effect of cyclosporine A) was the most common TEAE observed with canagliflozin and cyclosporine A coadministration (83.3%; 15/18). 

No deaths, serious TEAEs, or discontinuations due to TEAEs were reported in any of the studies. No consistent, clinically relevant changes for any of the clinical laboratory values, vital signs, physical examinations, or ECGs were noted. 

## Discussion 

Canagliflozin is primarily metabolized via UGT1A9 and UGT2B4 to form two major, pharmacologically-inactive glucuronidated metabolites, M5 and M7, which are excreted in the urine (~ 33%); less than 1% of the canagliflozin dose is excreted in urine as unchanged canagliflozin [6, 12, 36]. Canagliflozin is excreted by a balanced renal and biliary pathway [6, 36]. In addition, canagliflozin is a substrate of P-gp and MRP2. The 300 mg q.d. canagliflozin dose used in the current Phase-1 studies described here was also the maximum dose evaluated in the canagliflozin Phase-3 studies. 

Induction of metabolic clearance of a drug by co-medications can lead to decreased systemic exposure of the parent drug, decreasing its therapeutic effect [37]. In study 1, the recommended clinical dose of rifampin 600 mg q.d. was used [38]. Because the drug-metabolizing enzymes are known to be induced completely after 7 days of treatment with rifampin [39], canagliflozin was administered on day 10 (rifampin was administered from day 4 to 9). Rifampin, a potent and non-specific inducer of drug-metabolizing enzymes and transporters, decreased the C_max_ and AUC_∞_ of canagliflozin by 28% and 51%, respectively. The decreased systemic exposures of canagliflozin, in combination with the increased C_max_ of M5 and M7 after coadministration with rifampin, are consistent with the induction of both UGT1A9 (expressed in liver, kidney, and gastrointestinal tract) and UGT2B4 (expressed in liver and in multiple extrahepatic tissues, including heart). Because canagliflozin is a substrate of P-gp and MRP2, the reduction in canagliflozin exposure may be due to rifampin-induced increases in P-gp and MRP2 expression, leading to enhanced gut effect or biliary excretion. As a consequence of UGT1A9 induction, the plasma AUC_∞_ of M7 and urinary recovery were expected to increase, and not decrease as seen in this study. The observed decrease in M7 plasma AUC_∞_ (by 32%) and urinary recovery, in the absence of altered CL_R_, suggests that the transporter(s) involved in the biliary excretion of M7 may have been induced by rifampin. Similarly, the much smaller increase in M5 plasma AUC_∞_ (by 4%) relative to that in M5 plasma C_max_ (by 61%) following coadministration with rifampin, in the absence of altered CL_R_, also suggests that the transporter(s) involved in the biliary excretion of M5 may have been induced by rifampin. Since CYP3A4 plays a minimal role in canagliflozin metabolism [14], it is also possible that induction of CYP3A4 by rifampin may have contributed in part to the observed decrease in canagliflozin plasma exposures. 

Coadministration with rifampin decreased canagliflozin exposure, which may decrease the efficacy of canagliflozin. If a combined inducer of these UGT enzymes and drug transporters (e.g., rifampicin, phenytoin, barbiturates, phenobarbital, ritonavir, carbamazepine, efavirenz, St John’s wort (*Hypericum perforatum*)) must be coadministered in a patient receiving 100 mg q.d. canagliflozin, glycosylated hemoglobin (HbA_1c_) should be monitored with consideration to increasing the canagliflozin dose to 300 mg q.d. if additional glycemic control is needed. 

For patients receiving the 300 mg q.d. dose, a 50% reduction in canagliflozin exposure is still predicted to provide plasma exposures and HbA_1c_ lowering efficacy that is slightly greater than those in patients receiving 100 mg q.d. canagliflozin [40, 41, 42]. Based on these observations, drug-drug interactions that lead to reductions in canagliflozin exposures of less than 50% are not predicted to lead to a loss of glycemic control for participants receiving the 300 mg dose. 

Comedications that inhibit metabolic clearance of a drug can lead to higher systemic exposures of the parent drug, leading to increased adverse events (AEs) at administered therapeutic doses [37, 43]. In study 2, a clinically recommended dose (500 mg b.i.d.) of probenecid was used [44, 45] for 3 days to assess the effects of multiple-dose probenecid on the steady state pharmacokinetics of canagliflozin. It has been noted in clinical studies that canagliflozin acutely increases urinary uric acid excretion by up to 2-fold [46]; however, with multiple dosing for 2 weeks, urinary uric acid excretion diminishes back to pre-treatment baseline levels [47]. Hence, canagliflozin was administered alone for the first 2 weeks in study 2 to ensure that urinary uric acid excretion was close to normal baseline levels, and a possible additive uricosuric effect of canagliflozin with probenecid coadministration, could be avoided. 

Coadministration of probenecid, a nonspecific inhibitor of several UGT enzymes and drug transporters [23], with canagliflozin (300 mg q.d.) resulted in slightly increased mean C_max.ss_ (13%) and systemic exposure (21%) of canagliflozin. These small increases in canagliflozin plasma exposures are not considered to be clinically important since canagliflozin 300 mg b.i.d. doses, that achieve plasma C_max.ss_ and AUC_τ.ss_ of 32% and 93% higher than the 300 mg q.d. dose (evaluated in this study), have been well-tolerated in prior clinical studies, including a 2-week multiple-dose study [13] and a 12-week dose-ranging study in T2DM participants [41]. 

Canagliflozin is a substrate of MRP2 and is not a substrate for other transporters inhibited by probenecid, such as OATP1B1 or organic anion transporter families (OAT1, OAT3). The increase in plasma canagliflozin C_max.ss_ and AUC_τ.ss_ in the presence of probenecid was small and may likely be due to the inhibition of UGT(s) and transporters. Coadministration of probenecid decreased the CL_R_ of both M5 and M7 (by 82% and 58%, respectively) and their urinary excretion (by 72% and 42%, respectively). The effect of probenecid on M5 and M7 cannot be explained solely by probenecid-induced UGT inhibition as the mean M/P ratios for C_max_ and AUC for both M5 and M7 increased with probenecid, suggesting that inhibition of renal and biliary transport of these metabolites by probenecid may contribute to these findings. In vitro studies have confirmed that M5 and M7 are not the substrates of transporters OATP1B1 and OAT1; however, the metabolites were not assessed for MRP2 and OAT3. Because canagliflozin undergoes glucuronidation by 2 different UGT enzymes, and glucuronidation is a high-capacity/low-affinity system, clinically relevant interactions of other drugs on canagliflozin PK via inhibition of glucuronidation are unlikely to occur. Because the increase in canagliflozin exposure is small in the presence of probenecid, there is no dose adjustment recommended with probenecid or other UGT inhibitors. 

In study 3, a 400 mg dose of cyclosporine A is considered to be adequate to evaluate a potential P-gp inhibition-based drug interaction, as based on a previous study with aliskiren, a known P-gp substrate [35]. Cyclosporine A was administered 30 minutes before canagliflozin administration to allow for maximal P-gp inhibitory effect on canagliflozin absorption. The mean (SD) blood cyclosporine A concentration at 2.5 hours after coadministration of cyclosporine A (400 mg) with canagliflozin was 1,375 (321) ng/mL, which is consistent with the expected level for cyclosporine A considered adequate to evaluate a potential P-gp inhibition-based drug interaction [35]. 

Coadministration of canagliflozin with a single dose of cyclosporine A increased the AUC_τ.ss_ of canagliflozin by 23%, while the C_max_ was unaffected. These small increases in canagliflozin plasma exposures are not considered to be clinically important as 300 mg b.i.d. doses have been well-tolerated in earlier studies [13, 41]. The absence of an effect on C_max_, and a small increase in systemic exposure of canagliflozin in the presence of cyclosporine A, suggested limited involvement of P-gp, MRP2, and CYP3A4 in the disposition of canagliflozin. Hence, no meaningful interactions would be expected with other inhibitors of P-gp. Therefore, no dose adjustment for canagliflozin during coadministration appears to be warranted. 

Canagliflozin was well-tolerated when administered either alone or in combination with rifampin, probenecid, and cyclosporine A. The TEAEs were either mild or moderate in intensity. No serious TEAEs or adverse changes in clinical laboratory test values or vital signs were seen. 

## Conclusion 

These pharmacokinetic interaction studies have demonstrated that coadministration of cyclosporine A and probenecid had no clinically relevant effect on the pharmacokinetics of canagliflozin, but coadministration with rifampin modestly reduced canagliflozin plasma concentrations. Coadministration of canagliflozin with rifampin, probenecid, and cyclosporine A was generally well-tolerated. 

## Acknowledgments 

The authors thank Mr. Shreekant Sharma (SIRO Clinpharm Pvt. Ltd.) for writing assistance and Dr. Wendy Battisti (Janssen Research and Development, LLC) for additional editorial assistance for the development of this manuscript. The authors also thank Dr. Jayalakshmi Natarajan for providing additional statistical support and guidance. The authors also thank the study participants of the three studies, without whom the studies would never have been accomplished, and the investigators for their participation in the studies: United States of America: Eleanor Lisbon, MD, and Kelli Craven, MD; Antwerp: Dr. Lien Gheyle, MD. 

## Conflict of interest 

The studies presented in this report were supported by Janssen Research and Development, LLC. All authors are employees of Janssen Research and Development, LLC or of Janssen Research and Development, a division of Janssen Pharmaceutica NV and hold stocks in the company, except Joe Murphy. The sponsor also provided a formal review of this manuscript. 

**Table 1.** Participant disposition, demographic, and baseline characteristics.

Characteristics	Study 1 (n = 14)	Study 2 (n = 14)	Study 3 (n = 18)
Age (years), mean (SD)	30.3 (8.44)	29.1 (9.71)	42.1 (9.57)
Sex, n (%)			
Men	14 (100)	13 (93)	15 (83)
Women	0 (0)	1 (7)	3 (17)
Race, n (%)			
Black or African-American	3 (21)	9 (64)	–
White	11 (79)	5 (36)	18 (100)
Ethnicity, n (%)			
Hispanic or Latino	2 (14)	2 (14)	–
Not Hispanic or Latino	12 (86)	12 (86)	18 (100)
Weight (kg), mean (SD)	75.0 (14.06)	79.6 (11.19)	80.5 (9.29)
Height (cm), mean (SD)	176.8 (4.76)	176.9 (7.49)	177.1 (8.62)
BMI (kg/m^2^), mean (SD)	23.9 (3.71)	25.4 (2.73)	25.7 (2.38)
Participants who completed the study	14	11	18
Withdrawn			
Lost to follow-up^a^	–	1	–
Protocol violation	–	1	–
Physician decision	–	1	–

BMI = body mass index; SD = standard deviation.^a^This participant was considered to have completed the study per protocol as he had completed all required assessments of the open-label phase and was included in both the pharmacokinetic and statistical analyses. Note: Percentages calculated with the number of participants in each group as denominator.

**Table 2.** Pharmacokinetic parameters of canagliflozin, M7, and M5 after administration of canagliflozin alone or with rifampin in healthy participants.

Canagliflozin (300 mg q.d.) and rifampin (600 mg q.d.)					
Canagliflozin					
Parameter	Arithmetic mean (SD)		Geometric mean^a^		
	Canagliflozin alone (n = 14)	Canagliflozin + rifampin (n = 14)	Canagliflozin alone (reference) (n = 14)	Canagliflozin + rifampin (test) (n = 14)	Estimated ratio (test/reference), % (90% CI)
C_max_ (ng/mL)	2,474 (805)	1,732 (385)	2,358.29	1,692.05	71.75 (61.13; 84.21)
AUC_∞_ (ng×h/mL)	21,695 (6151)	10,489 (2535)	20,938.95	10,210.73	48.76 (43.69; 54.43)
t_max_ (h)^b^	2.00 (1.00 – 4.00)	1.79 (1.00 – 4.00)	–	–	–
t_1/2_ (h)	12.9 (2.42)	11.2 (3.22)	–	–	–
Ae (% dose)	0.578 (0.180)	0.257 (0.127)	–	–	–
CL_R_ (L/h)	0.0849 (0.0318)	0.0791 (0.0477)	–	–	–
M7					
C_max_ (ng/mL)	1,905 (943)	2340 (631)	1,722.15	2,258.19	131.13 (115.45; 148.93)
AUC_∞_ (ng×h/mL)	20,771 (10,728)	13,390 (4,560)^c^	18,820.25^c^	12,716.53^c^	67.57 (60.52; 75.44)
t_max_ (h)^b^	3.50 (2.00 – 6.00)	3.00 (2.00 – 4.00)	–	–	–
t_1/2_ (h)	13.3 (2.65)	10.7 (5.23)^c^	–	–	–
% Ae (dose)	20.1 (5.72)	12.8 (2.75)	–	–	–
CL_R_ (L/h)	4.62 (1.62)	4.33 (1.50)	–	–	–
M/P C_max_ ratio	0.556 (0.170)	1.02 (0.328)	–	–	–
M/P AUC_∞_ ratio	0.687 (0.233)	0.950 (0.319)^c^	–	–	–
M5					
C_max_ (ng/mL)	2,420 (1196)	3,616 (1095)	2,161.41	3,473.25	160.69 (134.22; 192.38)
AUC_∞_ (ng×h/mL)	21,903 (9556)	23,122 (8944)^c^	20,671.19^c^	21,522.27^c^	104.12 (92.83; 116.78)
t_max_ (h)^b^	4.00 (3.00 – 10.00)	4.00 (2.00 – 4.00)	–	–	–
t_1/2_ (h)	12.4 (2.43)	13.1 (3.12)^c^	–	–	–
% Ae (dose)	9.23 (3.41)	8.67 (2.79)	–	–	–
CL_R_ (L/h)	1.87 (0.559)	1.73 (0.589)	–	–	–
M/P C_max_ ratio	0.707 (0.240)	1.55 (0.413)	–	–	–
M/P AUC_∞_ ratio	0.733 (0.233)	1.58 (0.502)^c^	–	–	–

%Ae = cumulative amount excreted into the urine, calculated as (Ae/dose)×100, and corrected for molecular weight for M7 and M5; AUC_∞_ = area under the plasma concentration-time curve from time 0 to infinite time; C_max_ = maximum observed plasma concentration; CI = confidence interval; CL_R_ = renal clearance, calculated as: Ae_0–72_/AUC_0–72h_; M/P AUC_∞_ Ratio = metabolite-to-parent ratio for AUC_∞_; M/P C_max_ Ratio = metabolite-to-parent ratio for C_max_; q.d. = once daily; SD = standard deviation; t_1/2_ = elimination half-life; t_max_ = time to reach the maximum observed plasma concentration. ^a^Data analyzed on a logarithmic scale, but results transformed back to original scale; ^b^represented as median (range); ^c^n = 13.

**Table 3.** Pharmacokinetic parameters of canagliflozin, M7 and M5 after administration of canagliflozin alone or with probenecid in healthy participants.

Canagliflozin (300 mg q.d.) and probenecid (500 mg b.i.d.)					
Canagliflozin					
Parameter	Arithmetic mean (SD)		Geometric mean^a^		
	Canagliflozin alone (n = 12)	Canagliflozin + probenecid (n = 12)	Canagliflozin alone (reference)(n = 11)	Canagliflozin + probenecid (test) (n = 11)	Estimated ratio (test/reference), % (90% CI)
C_max.ss_ (ng/mL)	2,699 (814)	3,105 (680)	2,653.85	3,008.66	113.37 (100.37; 128.06)
AUC_∞.ss_ (ng×h/mL)	21,861 (4290)	26,225 (5261)	21,543.95	26,011.71	120.74 (116.37; 125.27)
t_max.ss_ (h)^b^	1.92 (1.42– 3.92)	1.67 (1.42 – 5.92)	–	–	–
% Ae (dose)	0.609 (0.217)	0.495 (0.136)	–	–	–
CL_R_ (L/h)	0.0868 (0.0297)	0.0569 (0.00944)	–	–	–
M7					
C_max.ss_ (ng/mL)	1,676 (611)	2,299 (811)	1,644.65	2,114.68	128.58 (120.37; 137.35)
AUC_∞.ss_ (ng×h/mL)	16,480 (5661)	22,405 (7746)	16,143.27	21,004.83	130.12 (126.11; 134.25)
t_max.ss_ (h)^b^	2.92 (1.92 – 3.92)	2.92 (1.92 – 5.92)	–	–	–
% Ae (dose)	21.2 (4.13)	12.2 (2.40)	–	–	–
CL_R_ (L/h)	5.80 (1.91)	2.46 (0.995)	–	–	–
M/P C_max.ss_ ratio	0.479 (0.190)	0.564 (0.220)	–	–	–
M/P AUC_∞.ss_ ratio	0.579 (0.238)	0.652 (0.249)	–	–	–
M5					
C_max.ss_ (ng/mL)	2,448 (805)	3,164 (934)	2,363.34	3,059.42	129.45 (116.45; 143.91)
AUC_τ.ss_ (ng×h/mL)	20,789 (7928)	30,607 (11801)	19,743.25	28,890.54	146.33 (135.09; 158.50)
t_max.ss_ (h)^b^	2.92 (2.92 – 3.92)	3.92 (1.92 – 5.92)	–	–	–
% Ae (dose)	11.2 (2.74)	3.09 (0.943)	–	–	–
CL_R_ (L/h)	2.47 (0.709)	0.454 (0.197)	–	–	–
M/P C_max.ss_ ratio	0.695 (0.249)	0.765 (0.217)	–	–	–
M/P AUC_τ.ss_ ratio	0.714 (0.286)	0.862 (0.284)	–	–	–

%Ae = cumulative amount excreted into the urine, calculated as (Ae/dose)×100, and corrected for molecular weight when necessary; AUC_τ.ss_ = area under the plasma concentration-time curve during a dosing interval at steady state; b.i.d. = twice daily; C_max.ss_ = maximum observed plasma concentration during a dosing interval at steady state; CI: confidence interval; CL_R_ = renal clearance, calculated as Ae_24_/AUC_24_; M/P AUC_τ.ss_ ratio = metabolite-to-parent ratio for AUC_τ.ss_; M/P C_max.ss_ ratio = metabolite-to-parent ratio for C_max.ss_; q.d. = once daily; SD = standard deviation; t_max.ss_ = time to reach the maximum observed plasma concentration during a dosing interval at steady state; τ = 24 hours interval. ^a^Data analyzed on a logarithmic scale, but results transformed back to original scale; ^b^represented as median (range).

**Table 4.** Pharmacokinetic parameters of canagliflozin after administration of canagliflozin alone or with cyclosporine A in healthy participants.

Canagliflozin (300 mg q.d.) and cyclosporine A (400 mg q.d.)					
Parameter	Arithmetic mean (SD)		Geometric mean^a^		
	Canagliflozin alone (n = 18)	Canagliflozin + cyclosporine A (n = 17)	Canagliflozin alone (reference) (n = 17)	Canagliflozin + cyclosporine A (test) (n = 17)	Estimated ratio (test/reference), % (90% CI)
C_max.ss_ (ng/mL)	2,887 (735)	2,923 (467)	2,864.03	2,887.13	100.81 (91.31; 111.30)
AUC_τ.ss_ (ng×h/mL)	22,158 (4203)	27,819 (5267)	22,243.50	27,355.78	122.98 (118.66; 127.46)
t_max.ss_ (h)^b^	2.00 (1.00 – 3.00)	4.00 (1.50 – 6.00)	–	–	–

AUC_τ.ss_ = area under the plasma concentration-time curve during a dosing interval at steady state; C_max.ss_ = maximum observed plasma concentration during a dosing interval at steady state; CI = confidence interval; q.d. = once daily; SD = standard deviation; t_max.ss_ = time to reach the maximum observed plasma concentration during a dosing interval at steady state; τ = 24 hours interval. ^a^Data analyzed on a logarithmic scale, but results transformed back to original scale; ^b^represented as median (range).
